# Transcatheter aortic valves produce unphysiological flows which may contribute to thromboembolic events: An in-vitro study

**DOI:** 10.1016/j.jbiomech.2016.10.050

**Published:** 2016-12-08

**Authors:** Andrea Ducci, Francesco Pirisi, Spyridon Tzamtzis, Gaetano Burriesci

**Affiliations:** a UCL Cardiovascular Engineering Laboratory, UCL Mechanical Engineering, University College London, UK; b Fondazione Ri.MED, Bioengineering Group, Palermo, Italy

**Keywords:** Transcatheter aortic valve implantation (TAVI), Blood stagnation, Particle Image Velocimetry (PIV), Thrombo-embolism, Valsalva sinus

## Abstract

**Purpose:**

Transcatheter aortic valve implantation (TAVI) has been associated with large incidence of ischemic events, whose sources are still unclear. In fact, sub-acute complications cannot be directly related to the severity of the calcification in the host tissues, nor with catheter manipulation during the implant. A potential cause could be local flow perturbations introduced by the implantation approach, resulting in thrombo-embolic consequences. In particular, contrary to the surgical approach, TAVI preserves the presence of the native leaflets, which are expanded in the paravalvular space inside the Valsalva sinuses. The purpose of this study is to verify if this configuration can determine hemodynamic variations which may promote blood cell aggregation and thrombus formation.

**Methods:**

The study was performed in vitro, on idealized models of the patient anatomy before and after TAVI, reproducing a range of physiological operating conditions on a pulse duplicator. The fluid dynamics in the Valsalva sinuses was analyzed and characterized using phase resolved Particle Image Velocimetry.

**Results:**

Comparison of the flow downstream the valve clearly indicated major alterations in the fluid mechanics after TAVI, characterized by unphysiological conditions associated with extended stagnation zones at the base of the sinuses.

**Conclusion:**

The prolonged stasis observed in the Valsalva sinuses for the configuration modelling the presence of transcatheter aortic valves provides a fluid dynamic environment favourable for red blood cell aggregation and thrombus formation, which may justify some of the recently reported thromboembolic and ischemic events. This suggests the adoption of anticoagulation therapies following TAVI, and some caution in the patients׳ selection.

## 1 Introduction

Aortic valve replacement is the definitive therapy for severe aortic stenosis (Bach et al., 2009), and is normally performed by open-heart surgical procedure, with the removal of the native valve and its replacement with a mechanical or a tissue valve. Transcatheter aortic valve implantation (TAVI) has recently emerged as a more suitable option for patients with severe symptomatic aortic stenosis, who are considered unsuitable for conventional surgery because of severe comorbidities, the principal risk factor being the age of patients. In this case, the valve substitute is delivered through the endovascular system via one of several access routes: transfemoral, transapical, subclavian and direct aortic (Bleiziffer et al., 2013). Clinical follow-up has documented a marked reduction in rehospitalisation with transfemoral TAVI as compared with medical management and, in comparison with surgery, a significantly shorter length of stay and earlier improvement in functional status (Reynolds et al., 2012, Smith et al., 2011). Also, transcatheter valves are commonly characterized by hydrodynamic performance, systolic pressure drop, equivalent or superior to surgically implanted valves, due to the thinner supporting frame and the absence of the sewing ring, which result into larger orifice areas (Smith et al., 2011, Webb and Cribier, 2010).

Conversely, TAVI is associated with new complications which include recurrence of mild to moderate paravalvular leakage, resulting in a less favourable late survival (Makkar et al., 2012); higher risk of injuries to the atrioventricular conduction system producing partial or complete heart blockage (Eltchaninoff et al., 2011, Moat et al., 2011); and high incidence of procedural and sub-acute ischemic events and subclinical leaflets thrombosis (Astarci et al., 2011, Kahlert et al., 2010, Makkar et al., 2015, Rodés-Cabau et al., 2010; Samim et al., 2015). Whilst the cause for the leakage and conduction impairments are well known and understood (Bagur et al., 2012, Lerakis et al., 2013), the reasons leading to post-implant thrombo-embolic consequences are still unclear. Nombela-Franco et al. (2012) reported that, in a large sample of 1061 patients, nearly half of 30-day ischemic events occurs after the first 24 h with rates of acute and sub-acute Cerebrovascular Events (CEVs), being 2.7% and 2.4%, respectively. Similarly, a recent review of TAVI studies/registries (including FRANCE II, PARTNER II, The European Sentinel, Advance) indicates that 30 days CEVs occur with 1.8–3.6% rates (Fanning et al., 2014). Recently, it has been suggested that a potential cause for post-procedural ischemic events occurring could be associated with local flow alterations (Colli et al., 2016, Ducci et al., 2013; Rodés-Cabau et al., 2013). This paper presents a study aimed at verifying the hemodynamic variations produced by the presence of the native aortic valve in the paravalvular space, and its potential contribution to thrombosis and subacute CEVs. The analysis is performed experimentally, on an idealized model, by means of Particle Image Velocimetry (PIV), and provides a qualitative and quantitative characterization of the fluid dynamics in the Valsalva sinus pre- and post-TAVI.

## 2 Methods

### 2.1 Pulse duplicator and cardiac chambers

Physiological hemodynamic conditions were reproduced in vitro using a cardiovascular pulse duplicator system (Vivitro Superpump System SP3891, Vivitro, Victoria, Canada). This is a hydro-mechanical mock circulatory system based on the Windkessel model, powered by a digitally controlled pump. The system includes view ports to observe valve function and transducer sites to measure flow and fluid pressure in all cardiac chambers.

A mock aortic root was made by casting from a two-part, optically clear, solvent free, low viscosity silicone elastomer (MED-6015, NuSil Technology, Carpinteria, CA, USA). The design of the root was based on the geometry and dimensions provided by Swanson and Clark (1974), Thubrikar (1990) and Reul et al. (1990), and had a diameter at the sino-tubular junction equal to 29 mm. A mock aortic valve fitting the root, based on the shape and dimensions reported by Thubrikar (1990) for a healthy human valve, was manufactured by dip coating, using a silicone dispersion (MED10-6607, Nusil, Carpinteria, CA, USA), with an average thickness of about 300 µm. The assembly of the described root and valve was used to model an idealized healthy physiological situation, and will be referred to as ‘reference configuration’ in the rest of this paper.

**Fig. 1:**
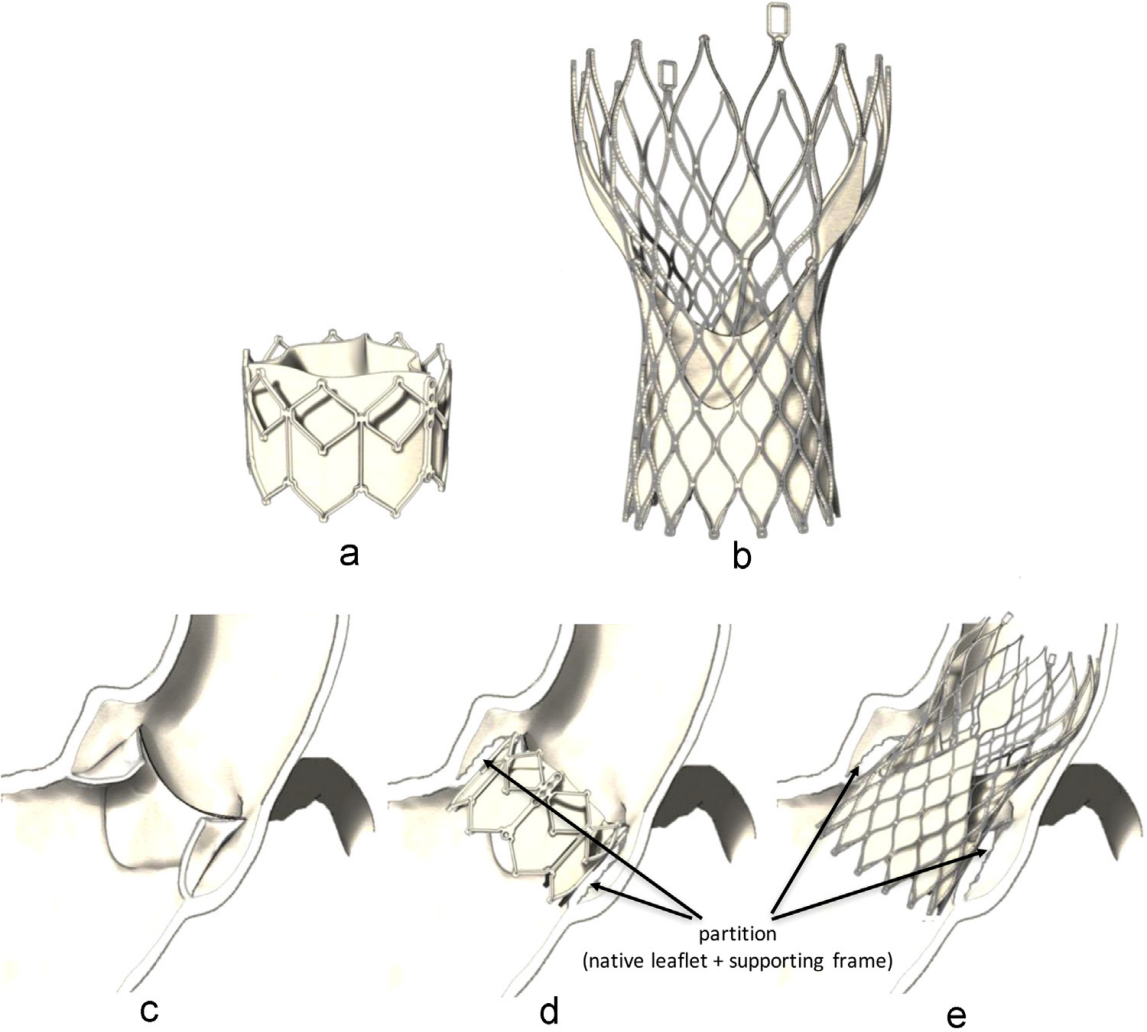
Sketches of the most widely used TAVI devices and of the aortic root before and after implantation: a) Edwards SAPIEN XT; b) CoreValve ReValving System; c) native aortic root; d) aortic root with Edwards SAPIEN XT; e) aortic root with CoreValve.

As previously described, the objective of this study was to investigate the changes produced on the fluid flow by the presence of the native valve leaflets after implantation of transcatheter aortic valves. Currently, there are two valve platforms in widespread clinical use: the Edwards SAPIEN (Edwards Lifesciences Inc.), which uses a balloon-expandable tubular frame made of cobalt-chromium alloy (stainless steel in the first versions), supporting three leaflets made from bovine pericardium and a PET fabric coating to provide an annular seal (Fig. 1a); and the CoreValve (Medtronic Inc.), which uses a self-expanding frame made of nickel–titanium alloy, supporting three leaflets and a sealing skirt made from porcine pericardium (Fig. 1b). A growing number of newer transcatheter valves are in clinical evaluation, but in all cases the prosthesis is implanted inside the leaflets of the native diseased valve (Fig. 1d and e), which are expanded by a tubular supporting frame and constrained to stay in this configuration, while the occluding function is provided by three prosthetic flexible membranes. In order to verify how TAVI alters the flow in a generalized case, a thin cylindrical transparent wall made of acetate, with thickness equal to 300 µm, was positioned around the leaflets of the valve, starting from their base and with a height equal to the length of the leaflet axial centreline. This corresponds to the most idealized post-implant condition, with: a) prosthetic leaflets identical to the ones of the reference native-like valve and implanted in the same orthotopic position; b) native leaflets of about constant thickness, presenting no calcification and expanded in a perfectly cylindrical fashion; and c) total absence of paravalvular leakage. This condition allows isolating the effect of the ‘valve-in-valve’ configuration on the flow, by avoiding any other spurious influence that could potentially contribute to affect the local flow dynamics (i.e. prosthetic and patient specific contributions). In the rest of this paper, this testing set-up will be referred to as ‘post-TAVI configuration’.

**Fig. 2:**
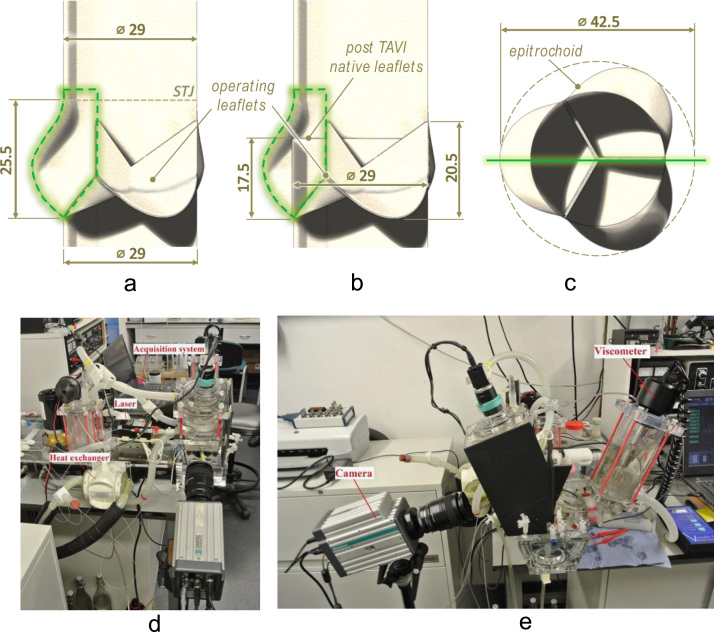
(a–c) Side and top view sketches of the mock valve-root systems, showing the main dimensions and the PIV measurement region; (d, e) Experimental set up.

Dimensions of the mock valve-root systems for the reference and post-TAVI configurations are provided in Fig. 2a–c.

Tests were performed at different combinations of physiological conditions, characterised by three beat rates of 60, 70 and 80 bpm and three cardiac outputs of 3, 4.5 and 6 l/min. In order to reduce the mechanical load acting on the closed valve, a mean systolic aortic pressure of 55 mmHg was used. Though this is lower than the normotensive value, which is around 100 mmHg, it was verified that the transvalvular systolic pressure differences and the flowrates are not significantly affected by these hypotensive conditions.

### 2.2 Particle Image Velocimetry

**Fig. 3:**
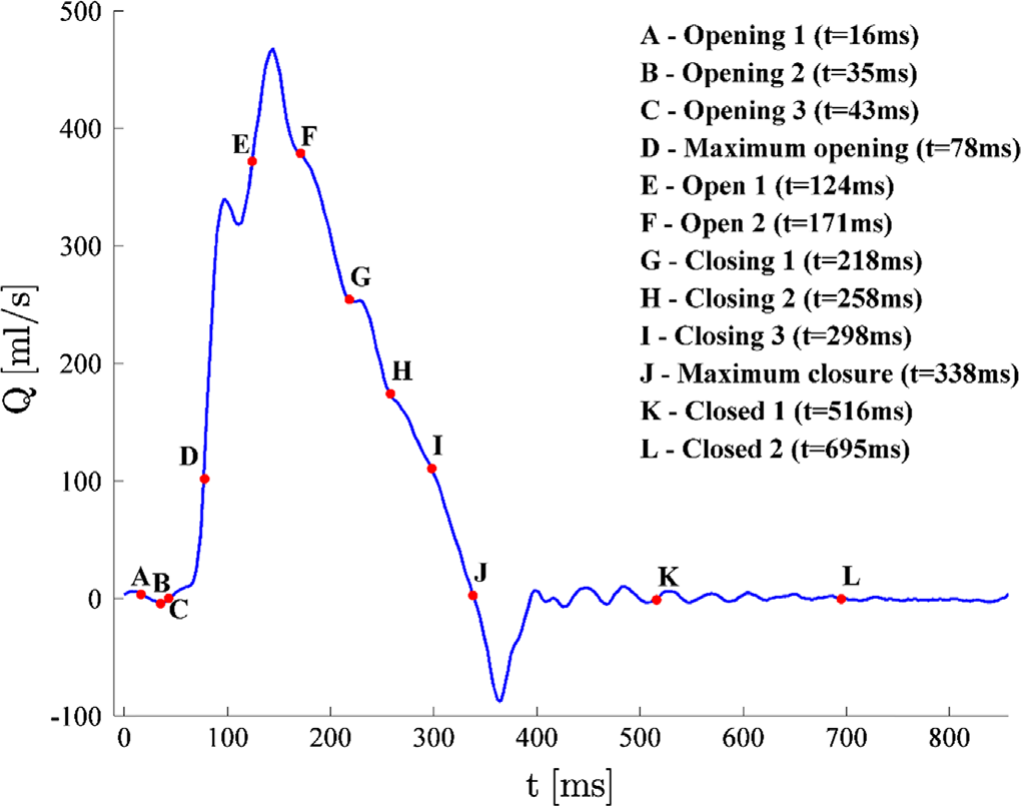
Typical diagram of the volumetric flowrate per cycle showing the selected time instants analysed with PIV (70 bpm and 4.5 l/min).

Flow characterisation was performed using 2D Particle Image Velocimetry (PIV). This is a non-intrusive optical technique, which provides measurements of the instantaneous velocity vector field in a plane located in the flow region of interest. The system employed in this study consisted of a continuous diode laser of 300 mW and wavelength of 532 nm, a cylindrical lens to convert the laser beam into a light sheet of approximately 1 mm thickness, and a high-speed intensified camera. The PIV system was synchronised with the pump of the pulse duplicator through a timing box, and the camera was triggered at selected time instants of the cardiac cycle to collect pairs of images Δ*t* = 3 ms apart. The experimental set up and measurement regions are provided in Fig. 2. For a thorough characterization of the flow dynamics of the cardiac cycle, 12 time instants were selected, as described in Fig. 3: points *A*, *B* and *C* during valve opening; point *D* at the maximum valve opening; points *E* and *F* after complete opening and before closing starts; points *G*, *H* and *I* during valve closing; point *J* at the at first instant after systole when the flow becomes equal to zero; and points *K* and *L* when the valve is closed. For each time instant phase resolved averages were obtained from a sample of 500 instantaneous velocity fields. Silver-coated hollow spherical particles of 10 µm average diameter and 1400 kg/m^3^ density were used, while the adaptive correlation algorithm built in Dynamics Studio (Dantec Ltd.) with a 50% window overlap was employed to obtain a final resolution of 8×8 pixels (corresponding to 0.1×0.1 mm). To best compare the phase resolved velocity fields associated with the different configurations analysed, the local velocity magnitude, *v*, was normalised with a reference velocity, *v*_*ref*_, defined as in Eq. (1):

(1) $$v_{\mathrm{ref}}=\frac{Q}{A_{\mathrm{STJ}}};$$

where *Q* is the volumetric flowrate and *A*_*STJ*_ is the area of the cross section of the aortic root at the sino-tubular junction. The normalised velocity, v*, is obtained as in Eq. (2):

(2) $$v^{*}=\frac{v}{v_{\mathrm{ref}}}.$$

To visualise the flow within the sinus and its variation for the reference and post-TAVI configurations, contour and vector maps of the local velocity were calculated for each instant investigated. A mask with white streamlines was sketched in the zone above the aortic valve, where the local fluid velocity is considerably higher than that occurring in the Valsalva sinuses and could not be quantified with the time interval Δ*t* selected in this study to characterise the region of interest. The streamlines are solely reported in this work as a visual aid to qualitatively represent the flow in the fast flow region closer to the valve exit, and are sketched based on the particle streaks observed in the PIV snapshots for the corresponding instant of the cardiac cycle.

Phase resolved turbulence contour maps were determined based on the Reynolds triple decomposition averages described in Reynolds and Hussain (1971).

### 2.3 Blood analogue liquid

A number of pure liquids and liquid solutions have been used in other studies to simulate blood, including pure water (Falahatpisheh and Kheradvar, 2012, Van Steenhoven and Van Dongen, 1979, Wendt et al., 2012), saline solution (Akutsu et al., 2008, Bottio et al., 2004, Ducci et al., 2013, Grigioni et al., 2000) and a water–glycerol solution (Azadani et al., 2009, Bullen and Rogers, 1968, Grigioni et al., 2003, Guivier-Curien et al., 2009, Kadem et al., 2005, Kadem et al., 2006, Lim et al., 1994, Lim et al., 1998, Stühle et al., 2011, Yoganathan et al., 1986). When using a laser-based technique such as PIV, it is essential to minimise the optical distortion due to the refraction occurring at the interface between different media in complex geometries. This has often been addressed by using more complex solutions, such as water–glycerol–sodium iodide (Browne et al., 2000, Leo et al., 2006), saline solution with glycerol (Balducci et al., 2004) and distilled water with potassium iodide (Toninato et al., 2016).

For all described cases, the approximation of Newtonian behaviour is accepted, neglecting the variation of dynamic viscosity which becomes significant at levels of shear rate inferior to 100 s^−1^ (Chien, 1970; Fedosov et al., 2011; Kim et al., 2012).

In the present study, a clear colourless solution with same dynamic viscosity as blood at 37 °C (*µ*_*b*_ = 4·10–3 Pa s) and minimum refractive index mismatch with the mock aortic root and valve׳s leaflets (*n* = 1.40) was developed by adding glycerol (37 vol%) to a solution of water and potassium iodide (24 wt%). To identify the optimum volumetric ratio of the mixture, providing best blood viscosity and refractive index matching, the correlation coefficient, *R_bw_*, was estimated according to Eq. (3):

(3) $$R_{\mathrm{bw}}=\frac{I_{d}\cdot I_{0}}{I_{0}\cdot I_{0}}$$

**Fig. 4:**
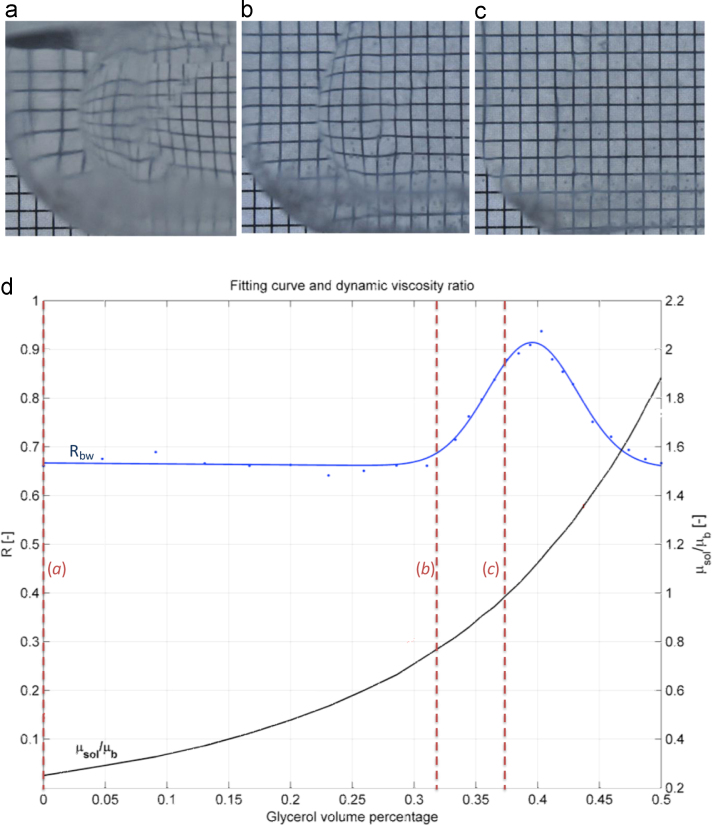
(a–c) Visualisation of the degree of distortion induced by refraction mismatch at the sinus wall, for glycerol volume percentage of: a) 0%; b) 32 %; c) 37 %; and, (d) variation of the correlation coefficient, *R*_*bw*_, and solution to blood viscosity ratio for increasing glycerol volume percentage.

This is based on the image projection of the distorted grid captured at the back of the root-fluid solution system, *I*_*d*_, on to an ideal grid corresponding to zero distortion, *I*_0_. Three characteristic images visualising different degree of optical distortion induced by the refraction at the sinus wall of the root are provided in Fig. 4a–c, for increasing glycerol percentage. From Fig. 4d, it can be seen that the solution provides minimum light distortion (maximum *R_bw_* coefficient) and dynamic viscosity, *μ_sol_*, similar to blood (*μ_sol_*/*μ_b_* ≈ 1) for a glycerol volumetric ratio of 37%.

## 3 Results

**Fig. 5:**
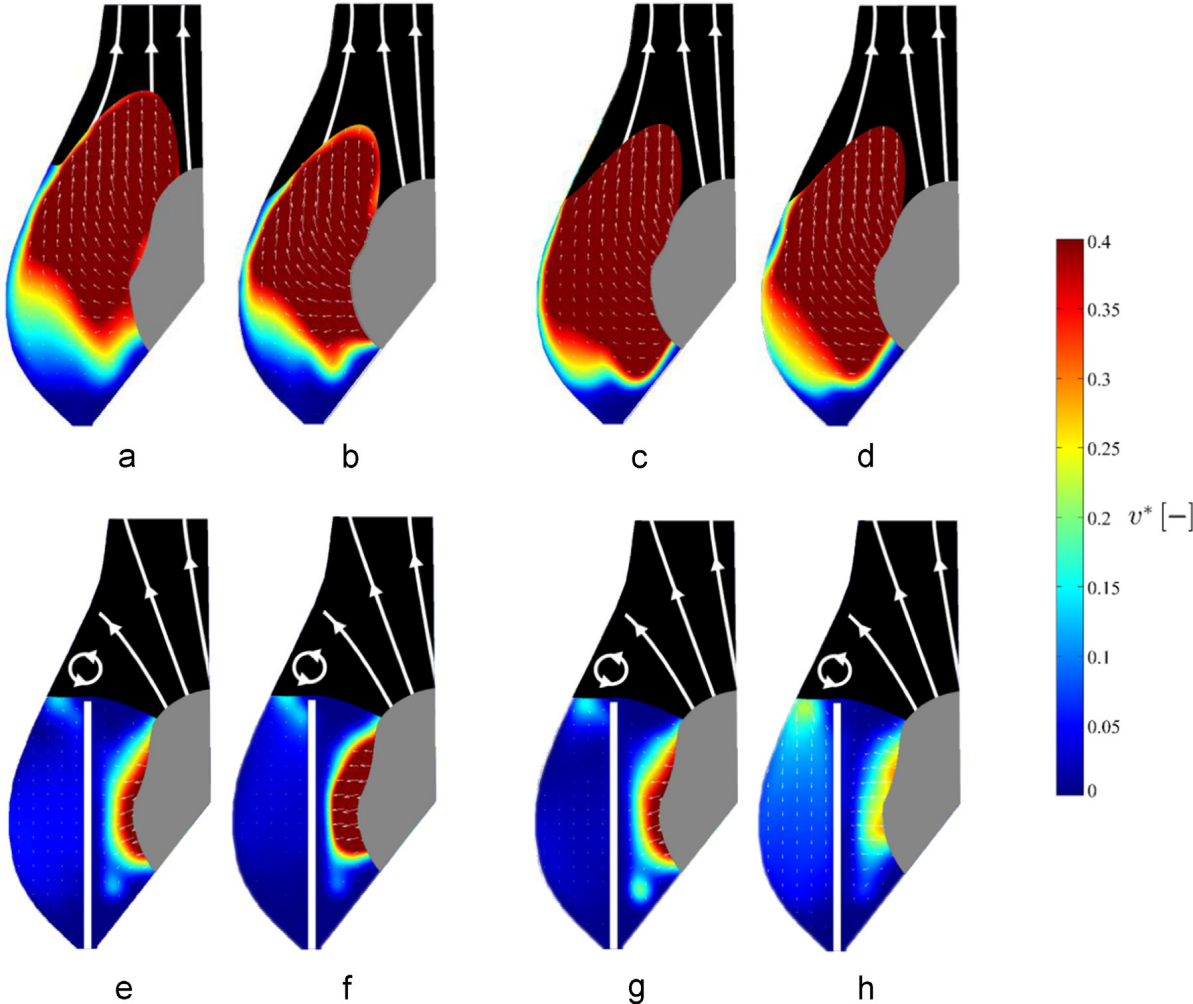
Flow fields of reference and post-TAVI configurations for different operating conditions at one representative instant of the opening phase (instant B) within the cardiac cycle: reference configuration at 60 bpm (a) and 4.5 l/min (b); reference configuration at 80 bpm (c) and 4.5 l/min (d); post-TAVI configuration at 60 bpm (e) and 4.5 l/min (f); post-TAVI configuration at 80 bpm (g) and 4.5 l/min (h).

The flow fields obtained for each configuration (reference and post-TAVI) at the different beat and flow rates were similar and presented identical features. This is evident in Fig. 5a–h, where characteristic flow fields associated to extreme cases (3–6 l/min, and 60–80 bpm) at instants *B* and *D* in the cardiac cycle exhibit similar flow patterns and scaled velocity intensity, both in pre- and post-TAVI configurations. Hence, for brevity, only the results corresponding to the intermediate condition (70 bpm and 4.5 l/min) are reported and discussed hereafter.

**Fig. 6:**
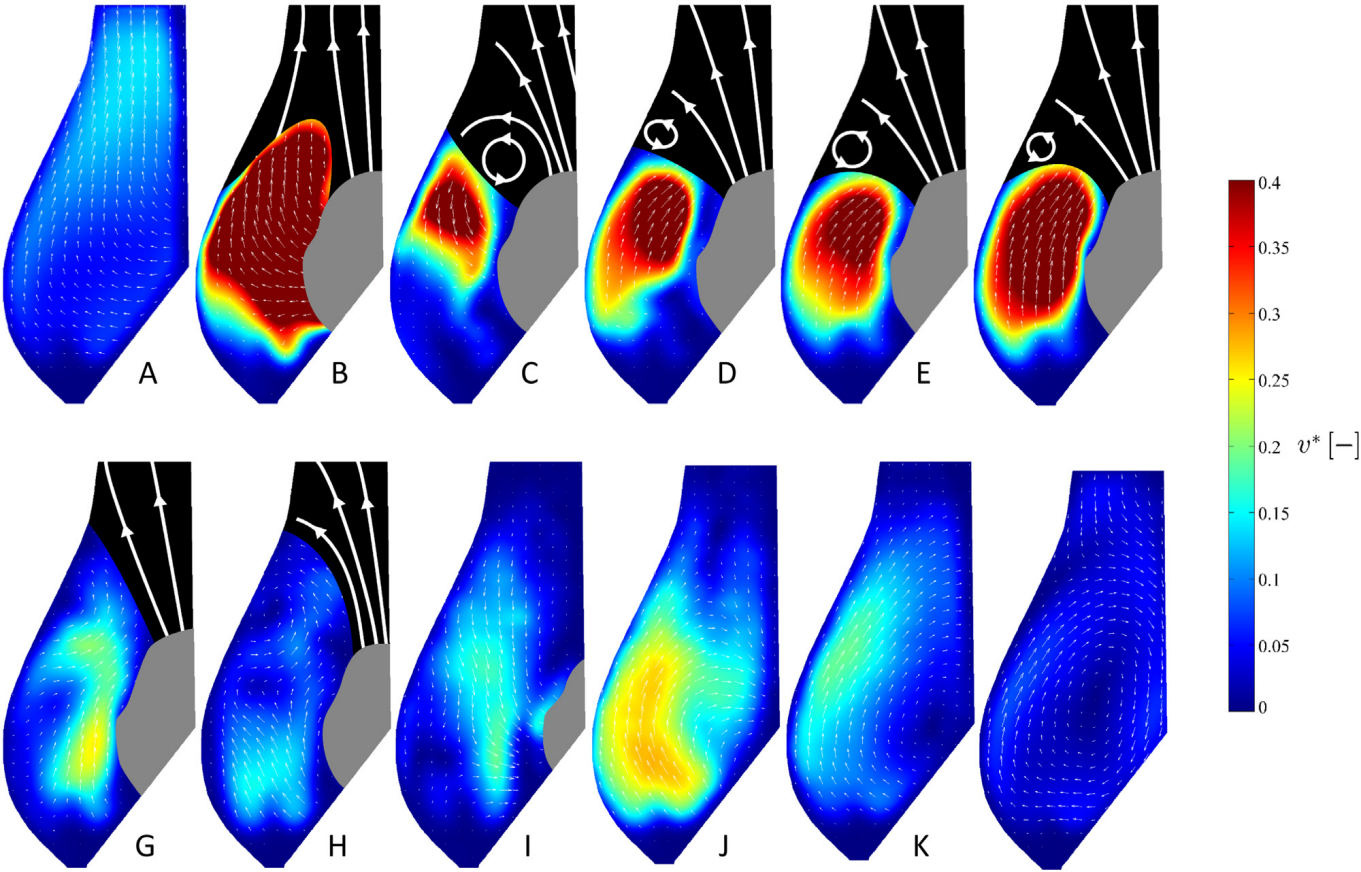
Phase resolved velocity contour map and vector fields for the reference configuration at different time instants throughout the cardiac cycle, for the representative intermediate operating conditions (70 bpm and 4.5 l/min).

Fig. 6 presents the phase resolved velocity maps for these intermediate conditions. At the beginning of the systole (instant *A*), the flow is directed radially next to the leaflet, then turns to follow the Valsalva׳s sinus shape, aligning with the vertical axis in the upper region, where the liquid leaves the aortic root. This flow pattern is qualitatively preserved for the entire opening phase, with local velocity magnitudes increasing fourfold at instant *B*. At instant *C*, after opening is complete, a vortex ring is formed at the top of the valve leaflet (counter-clockwise in the figure). From instant *C* to instant *F* this vortex moves radially towards the wall of the aortic root, at the top of the Valsalva׳s sinus, maintaining its direction of rotation and intensity. From instants *G* to *H* the ejection jet slows down, resulting in a reduction of the dimensionless velocity. The vortex ring is still visible, but its intensity has reduced significantly. Hence the valve starts closing (instants *H* to *J*), supported by a flow directed from the aortic root towards the leaflets. At instants *K* and *L* the valve is fully closed and a counter-rotating vortex (clockwise in the figure) develops and fills the entire sinus.

**Fig. 7:**
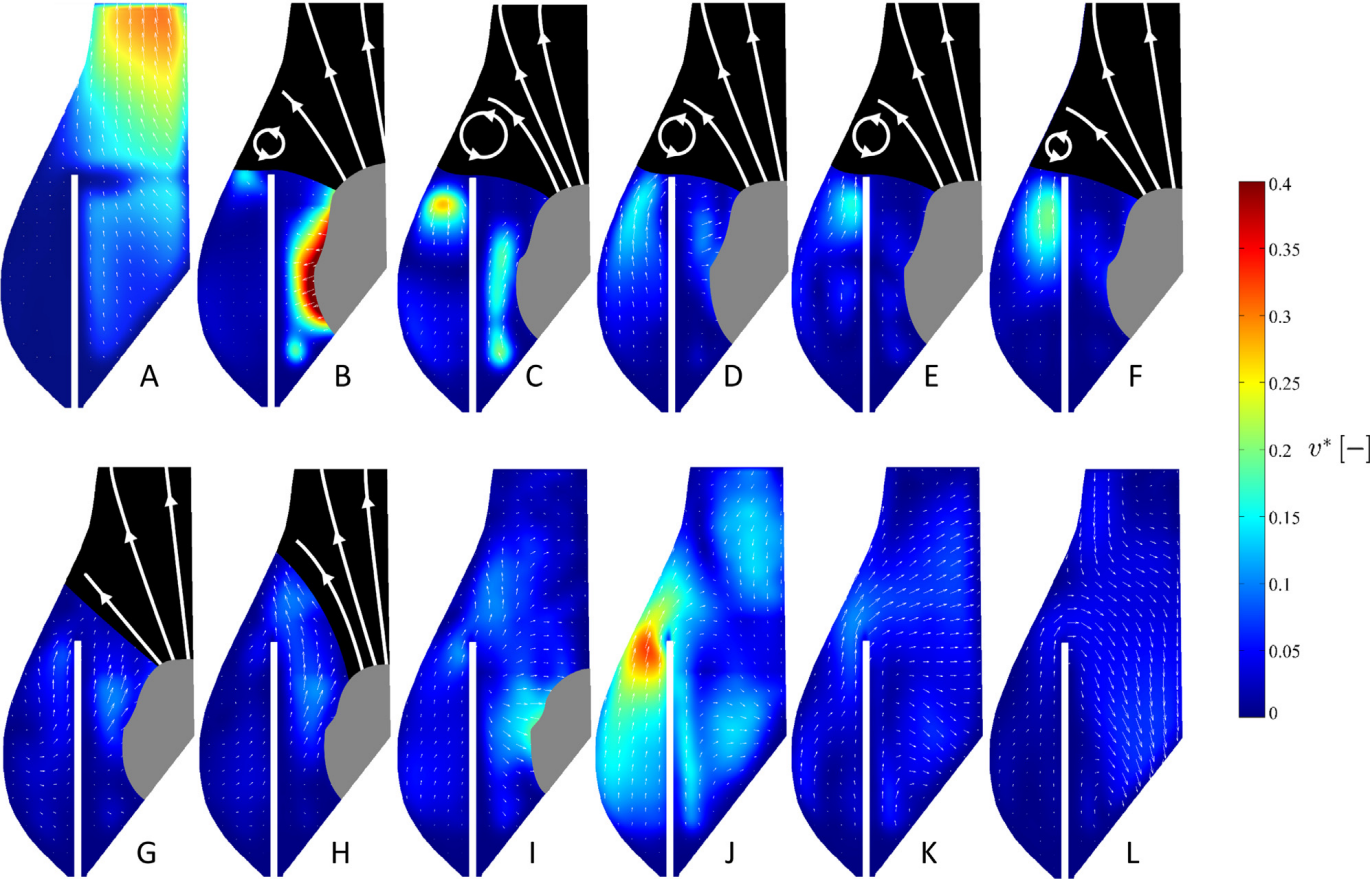
Phase resolved velocity contour map and vector fields for the post-TAVI configuration at different time instants throughout the cardiac cycle, for the representative intermediate operating conditions (70 bpm and 4.5 l/min).

The corresponding phase-resolved magnitude and velocity vector maps for the post-TAVI configuration at same operating conditions (70 bpm and 4.5 l/min) are provided in Fig. 7. Similarly to the physiological/reference configuration, at instant *A* the local flow direction is perpendicular to the leaflet in its proximity, and axial in the upper portion of the root. At instant *B*, as the leaflet moves towards its open position, a vortex ring forms at the exit of the cylindrical wall. The vortex remains in this region up to the instant of maximum flow (point *F*), but its intensity decreases as the flow velocity reduces (instants *G* to *H*). During valve closure, in instants *I* and *J*, the local depression created by the leaflet movement determines a fluid suction from the upper part of the Valsalva sinus, with fluid moving around the edge of the cylinder mimicking the presence of the native leaflets. This feature is still preserved during early diastole, as evident at instants *K* and *L*.

Direct comparison of the velocity fields of Fig. 6, Fig. 7 indicates that the post-TAVI configuration is associated with a substantial drop of flow velocity inside the Valsalva sinus, resulting in a zone of stagnation at the base of this region.

The described flow dynamic observed for the reference and post-TAVI configurations were consistently observed at all the other flow and heart beat rate examined, with no substantial variations in the dimensionless velocity. Obviously, the dimensional velocity increased proportionally with the flow rate.

**Fig. 8:**
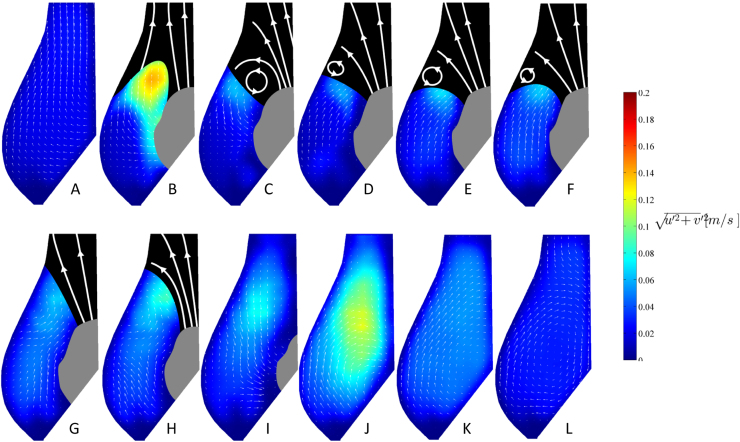
Phase resolved turbulence contour map and vector fields for the reference configuration at different time instants throughout the cardiac cycle, for the representative intermediate operating conditions (70 bpm and 4.5 l/min).

**Fig. 9:**
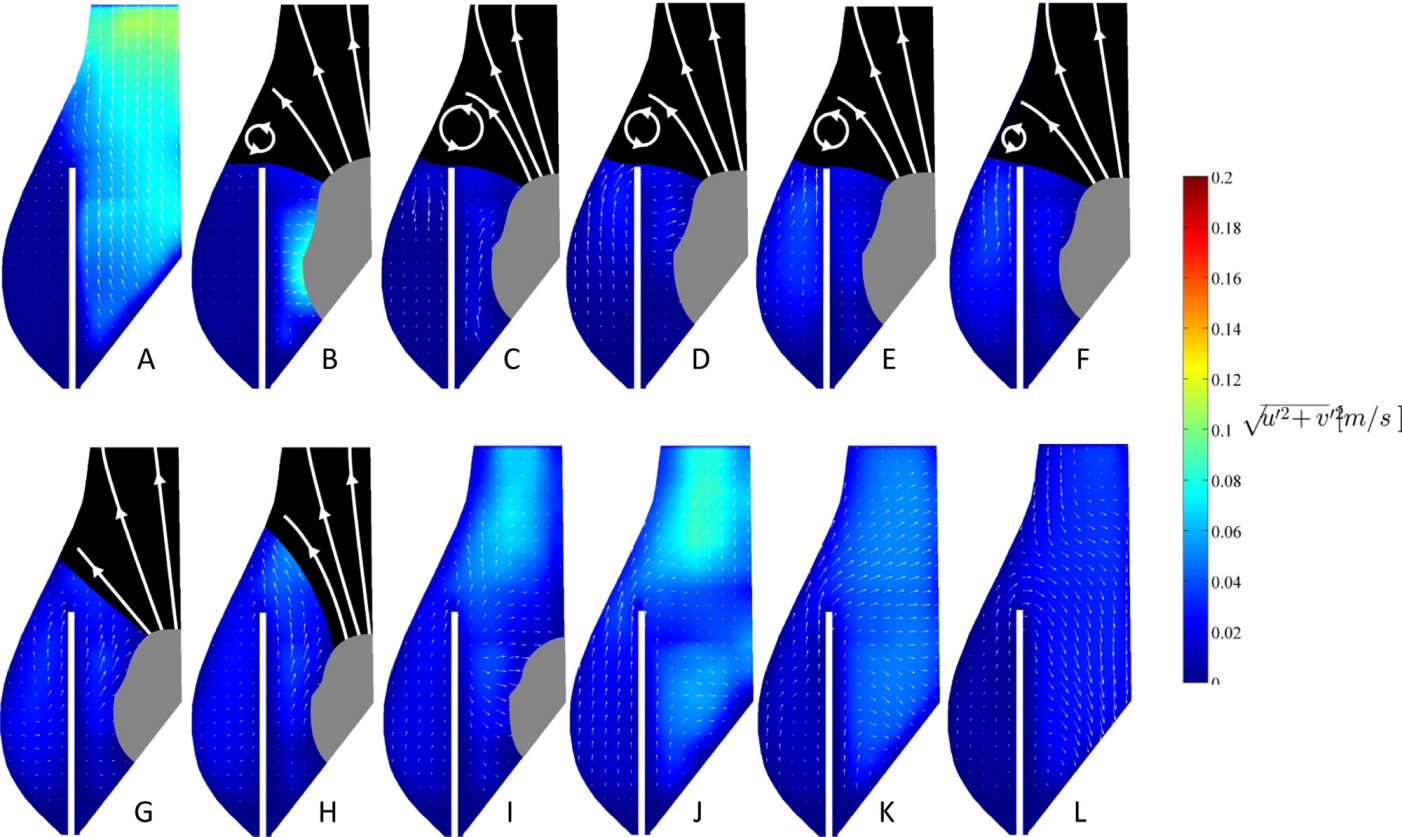
Phase resolved turbulence contour map and vector fields for the post-TAVI configuration at different time instants throughout the cardiac cycle, for the representative intermediate operating conditions (70 bpm and 4.5 l/min).

Analysis of the phase resolved turbulence levels in the Valsalva sinus, described in Fig. 8, Fig. 9, indicates that the reference configuration is associated with higher turbulence (0.15 vs. 0.10), but for both configurations the velocity standard deviation never exceed 0.2 m/s.

**Fig. 10:**
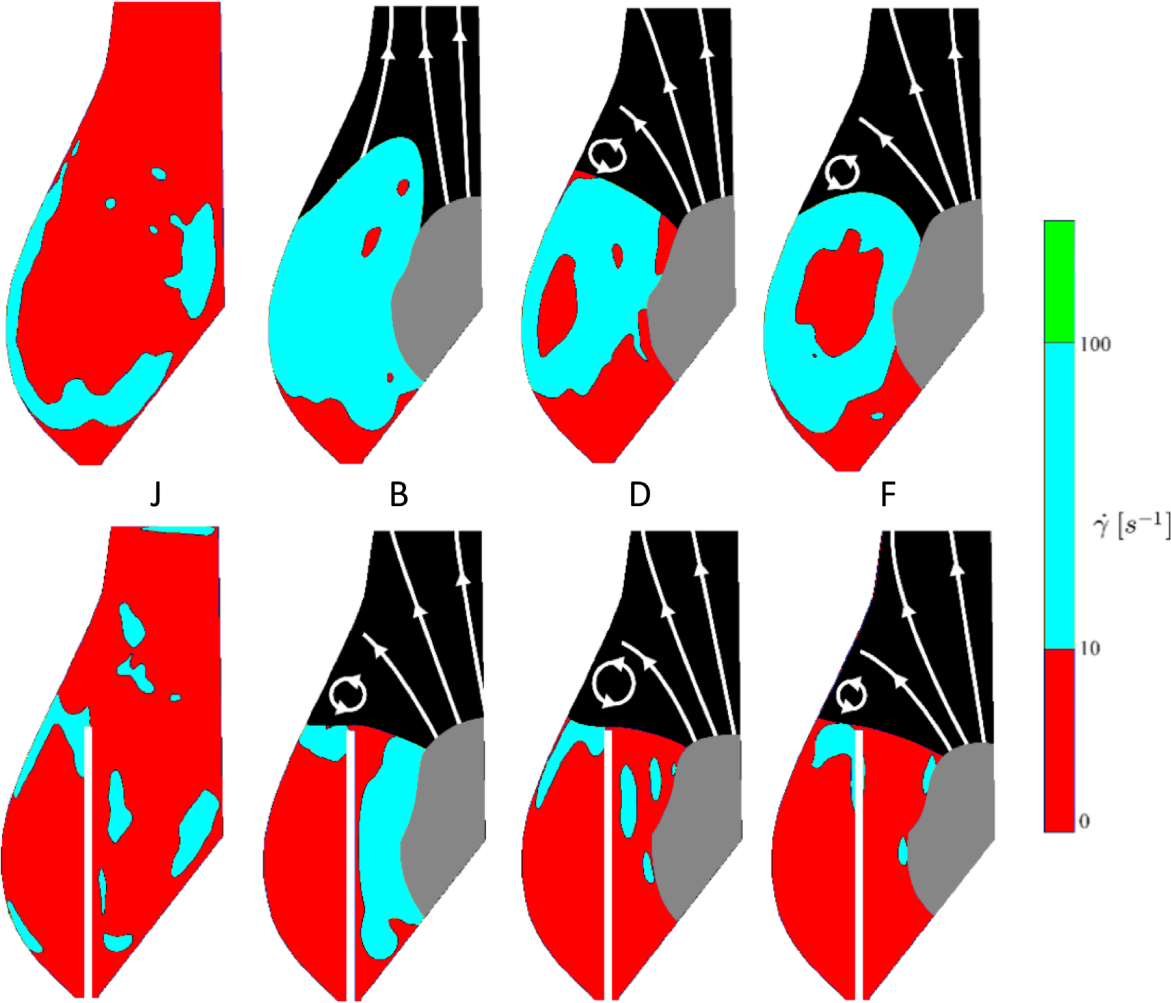
Visualisation of the flow regions subject to different degree of shear rate: (red) low shear rate, (cyan) intermediate shear rate, (green) high shear rate. Results for the reference and post-TAVI configurations are represented on the top and on the bottom row, respectively. (For interpretation of the references to colour in this figure legend, the reader is referred to the web version of this article.)

A comparison of the shear rate maps obtained in the Valsalva sinus for the two configurations before and after TAVI is provided in Fig. 10. Shear rate thresholds of 10 and 100 s^−1^ were selected to best visualize areas subject to high ($\dot{\gamma }$> 100 s^−1^), intermediate (10 < $\dot{\gamma }$< 100 s^−1^) and low ($\dot{\gamma }$< 10 s^−1^) shear, and to identify regions where Newtonian fluid assumption is valid (green threshold). In the post-TAVI configuration, the value of shear rate in the Valsalva sinus is lower than 10 s^−1^ over an extended region during the entire cardiac cycle. On the contrary, though regions of low shear rate are present during diastole also in the reference configuration, the sinus is periodically washed at each cardiac cycle, reducing the local residence time and preventing red blood cell aggregation (Kaliviotis and Yianneskis, 2008).

These dynamics are preserved for all the different combinations of beat rate and flow rate investigated, without significant variation of the velocity field, turbulence and shear rate.

## 4 Discussion

Comparison of the flow downstream the valve before and after TAVI clearly indicates major variations in the fluid mechanics and operating mechanisms of the valve. For the reference configuration, in the early stages of the opening the fluid runs along the Valsalva sinus wall with significant radial and axial velocity components. This motion supports the leaflet movement towards the open configuration. During this stage, a vortex ring forms at the edge of the valve׳s leaflet, and moves to the top of the Valsalva׳s sinus. The vortex stays in this position for the whole duration of the ejection phase and disappears when the leaflet moves to the closed position. When the valve is fully closed, a counter-vortex forms closed to the leaflet, and expands to the center of Valsalva׳s sinus.

In the case of the post-TAVI configuration, the fluid dynamics in the Valsalva sinus results substantially different. During opening, the leaflet of the native valve acts as a cylindrical wall, forcing an axial flow. As in the previous case, a vortex ring forms at the valve exit, at the top of the Valsalva sinus. However, this appears to be associated to the rigid native leaflets edge, rather than the operating prosthetic leaflets, and maintains its position for the whole duration of the ejection phase. The vortex disappears when the prosthetic leaflets move to the closed position. During closing, the liquid comes back to the leaflet in the axial direction, without producing the vortical structures observed for the native configuration. Between the sinus and the native leaflet partition develops an extended and prolonged stagnation zone with minimum shear rate (below 100 s^−1^), which is present during the full cycle. For a non-Newtonian fluid with the rheological behavior typical of human blood, this permanent level of shear rate leads to a substantial increase in the dynamic viscosity (Chien, 1970, Fedosov et al., 2011, Kaliviotis and Yianneskis, 2011, Kim et al., 2012). Hence, the prolonged stasis observed in the Valsalva sinus for the post-TAVI configuration is likely to be more severe in the in vivo condition, favouring red blood cell aggregation and thrombus formation.

This may justify some of the ischaemic complications reported after transcatheter heart valve implantation. In particular, though major strokes with permanent deficit are rare and have similar rate between TAVI and surgical replacements, as shown from studies at 30 days (Miller et al., 2012) and 2 years (Kodali et al., 2012), transcatheter approaches are associated with much higher rate of new post-procedural cerebral lesions, which are reported to affect up to 90% of treated patients (Astarci et al., 2011, Kahlert et al., 2010, Rodés-Cabau et al., 2010, Samim et al., 2015). The detected ischemic events are normally asymptomatic, but can be associated with unnoticed deficits in physical and cognitive functions, raising considerably the risk of successive stroke and dementia (Vermeer et al., 2007). There is no evidence that these embolic complication can directly be associated to the severity of the calcification present in the host tissues, as there is no significant correlation with the number and volume of the ischemic lesions (Rodés-Cabau et al., 2010, Samim et al., 2015). Also, catheter manipulation in the blood vessels or retrograde crossing of the aortic valve during the valve implantation does not appear to be a major cause for the lesions, as the incidence of embolic events is similar with the transfemoral approach, which reaches the aortic valve from the femoral artery, after crossing of the entire aorta, and the transapical access, which directly reaches the valve from the inflow, after puncturing the apex of the left ventricle (Astarci et al., 2011, Athappan et al., 2014, Rodés-Cabau et al., 2010).

This study shows that the presence of transcatheter aortic valves produces unphysiological flows in the aortic root that result into stagnation at the base of the Valsalva sinuses, supporting the hypothesis that the presence of the native aortic valve in the paravalvular space might promote blood stasis, which is a main contributing factor towards thrombosis and thromboembolic events (Ducci et al., 2013, Rodés-Cabau et al., 2013). This might also be reflected in some of the observed cases of post-TAVI thrombosis (De Marchena et al., 2015), which may be responsible for the higher incidence of reduced leaflet motion, recently reported by Makkar et al. (2015).

The findings from this study, which can provide some justification to the emerging clinical evidence, suggest the adoption of chronic anticoagulation therapies following TAVI, and recommend some caution when selecting this valve therapy for patients unsuitable for anticoagulant medications.

Of course, the interpretation of the present results has to take into consideration a number of assumptions, idealizations and limitations that were introduced in the experiments. Firstly, due to the attempt to obtain generalizable findings, the root and leaflets were based on idealized geometries, which do not consider patient specific irregularities. Moreover, they do not account for the coronary flow and the compliance of the aortic root. Though these factors are likely to reduce the blood stasis, the study can still be considered representative of the most common case in TAVI patients, where the aortic root is severely calcified, and indicative of the flow conditions in the posterior aortic sinus (non-coronary sinus), where the stagnation is expected to be more severe. Another approximation of the study is in the use of a Newtonian fluid, that does not model accurately the increase in viscosity occurring at low levels of shear stress, associated with the red cells aggregation. Finally, the PIV system set up was optimized to capture the slow flow motion occurring in the Valsalva sinus, and therefore could not resolve the fast flow regions occurring during systole.

## 5 Conclusions

This study presents a detailed analysis of the fluid dynamics inside the Valsalva sinus under ideal conditions before and after TAVI, identifying and quantifying the flow variation produced by the treatment. Quantification of relevant flow parameters was provided by means of phase resolved PIV measurements for a range of beat rates and cardiac outputs at selected time instants of the cardiac cycle.

The analysis of the flow downstream of the valve indicated that the fluid dynamics that occurs in physiological conditions into the Valsalva sinuses, and supports the opening and closing mechanisms, is largely altered when a TAVI device is implanted. In particular, a reduction of turbulence, velocity magnitude and shear rate is observed between the leaflets of the native valve and the aortic wall. This is associated with an extended stagnation zone at the base of the sinus, providing a fluid dynamic environment that may promote thrombotic phenomena and contribute to thromboembolic and ischaemic events.

## Conflict of interest

None of the authors has any relationship with industry or financial associations that might pose a conflict of interest in connection with this work.

## Disclosures

None.

## References

[bib1] Akutsu T., Saito J., Imai R., Suzuki T., Cao X.D. (2008). Dynamic particle image velocimetry study of the aortic flow field of contemporary mechanical bileaflet prostheses. J. Artif..

[bib2] Astarci P., Glineur D., Kefer J., D’Hoore W., Renkin J., Vanoverschelde J.L., El Khoury G., Grandin C. (2011). Magnetic resonance imaging evaluation of cerebral embolization during percutaneous aortic valve implantation: comparison of transfemoral and trans-apical approaches using Edwards Sapiens valve. Eur. J. Cardio-Thorac. Surg..

[bib3] Athappan G., Gajulapalli R.D., Sengodan P., Bhardwaj A., Ellis S.G., Svensson L., Tuzcu E.M., Kapadia S.R. (2014). Influence of transcatheter aortic valve replacement strategy and valve design on stroke after transcatheter aortic valve replacement: a meta-analysis and systematic review of literature. J. Am. Coll. Cardiol..

[bib4] Azadani A.N., Jaussaud N., Matthews P.B., Ge L., Guy T.S., Chuter T.A.M., Tseng E.E. (2009). Energy loss due to paravalvular leak with transcatheter aortic valve implantation. Ann. Thorac. Surg..

[bib5] Bach D.S., Siao D., Girard S.E., Duvernoy C., McCallister B.D., Gualano S.K. (2009). Evaluation of patients with severe symptomatic aortic stenosis who do not undergo aortic valve replacement the potential role of subjectively overestimated operative risk. Circ. Cardiovasc. Qual. Outcomes.

[bib6] Bagur R., Rodés-Cabau J., Gurvitch R., Dumont É., Velianou J.L., Manazzoni J., Toggweiler S., Cheung A., Ye J., Natarajan M.K., Bainey K.R., Delarochellière R., Doyle D., Pibarot P., Voisine P., Côté M., Philippon F., Webb J.G. (2012). Need for permanent pacemaker as a complication of transcatheter aortic valve implantation and surgical aortic valve replacement in elderly patients with severe aortic stenosis and similar baseline electrocardiographic findings. JACC Cardiovasc. Interv..

[bib7] Balducci A., Grigioni M., Querzoli G., Romano G.P., Daniele C., D’Avenio G., Barbaro V. (2004). Investigation of the flow field downstream of an artificial heart valve by means of PIV and PTV. Exp. Fluids.

[bib8] Bleiziffer S., Krane M., Deutsch M. a, Elhmidi Y., Piazza N., Voss B., Lange R. (2013). Which way in? The necessity of multiple approaches to transcatheter valve therapy.

[bib9] Bottio T., Caprili L., Casarotto D., Gerosa G. (2004). Small aortic annulus: the hydrodynamic performances of 5 commercially available bileaflet mechanical valves. J. Thorac. Cardiovasc. Surg..

[bib10] Browne P., Ramuzat A., Saxena R., Yoganathan A.P. (2000). Experimental investigation of the steady flow downstream of the St. Jude bileaflet heart valve: a comparison between laser Doppler velocimetry and particle image velocimetry techniques. Ann. Biomed. Eng..

[bib11] Bullen J.J., Rogers H.J. (1968). Effect of haemoglobin on experimental infections with Pasteurella septica and *Escherichia coli*. Nature.

[bib12] Chien S. (1970). Shear dependence of effective cell volume as a determinant of blood viscosity. Science.

[bib13] Colli A., Ducci A., Burriesci G. (2016). Possible subclinical leaflet thrombosis in bioprosthetic aortic valves. N. Engl. J. Med..

[bib14] De Marchena E., Mesa J., Pomenti S., Marin Y Kall C., Marincic X., Yahagi K., Ladich E., Kutz R., Aga Y., Ragosta M., Chawla A., Ring M.E., Virmani R. (2015). Thrombus formation following transcatheter aortic valve replacement. JACC: Cardiovasc. Interv..

[bib15] Ducci A., Tzamtzis S., Mullen M.J., Burriesci G. (2013). Hemodynamics in the Valsalva sinuses after transcatheter aortic valve implantation (TAVI). J. Heart Valve Dis..

[bib16] Eltchaninoff H., Prat A., Gilard M., Leguerrier A., Blanchard D., Fournial G., Iung B., Donzeau-Gouge P., Tribouilloy C., Debrux J.-L., Pavie A., Gueret P., FRANCE Registry Investigators (2011). Transcatheter aortic valve implantation: early results of the FRANCE (FRench Aortic National CoreValve and Edwards) registry. Eur. Heart J..

[bib17] Falahatpisheh A., Kheradvar A. (2012). High-speed particle image velocimetry to assess cardiac fluid dynamics in vitro: from performance to validation. Eur. J. Mech. – B/Fluids.

[bib18] Fanning J.P., Walters D.L., Platts D.G., Eeles E., Bellapart J., Fraser J.F. (2014). Characterization of neurological injury in transcatheter aortic valve implantation: how clear is the picture?. Circulation.

[bib19] Fedosov D., Pan W., Caswell B., Karniadakis G. (2011). Predicting human blood viscosity in silico. PNAS.

[bib20] Grigioni M., Daniele C., D’Avenio G., Barbaro V. (2000). Hemodynamic performance of small-size bileaflet valves: pressure drop and laser Doppler anemometry study comparison of three prostheses. Artif. Organs.

[bib21] Grigioni, M., Daniele, C., Del Gaudio, C., Morbiducci, U., Balducci, A., D’Avenio, G., Barbaro, V., 2003. Experimental and computational studies of flow through a bileaflet mechanical heart valve in a realistic aorta. Rapp. ISTISAN 03/27

[bib22] Guivier-Curien C., Deplano V., Bertrand E. (2009). Validation of a numerical 3-D fluid–structure interaction model for a prosthetic valve based on experimental PIV measurements. Med. Eng. Phys..

[bib23] Kadem L., Garcia D., Rieu R., Durand L.G., Pibarot P. (2005). Flow dynamics past a bioprosthetic valve using PIV and proper orthogonal decomposition. Proceedings of the Third IASTED International Conference on BIOMECHANICS.

[bib24] Kadem L., Rieu R., Dumesnil J.G., Durand L.-G., Pibarot P. (2006). Flow-dependent changes in Doppler-derived aortic valve effective orifice area are real and not due to artifact. J. Am. Coll. Cardiol..

[bib25] Kahlert P., Knipp S.C., Schlamann M., Thielmann M., Al-Rashid F., Weber M., Johansson U., Wendt D., Jakob H.G., Forsting M., Sack S., Erbel R., Eggebrecht H. (2010). Silent and apparent cerebral ischemia after percutaneous transfemoral aortic valve implantation: a diffusion-weighted magnetic resonance imaging study. Circulation.

[bib26] Kaliviotis E., Yianneskis M. (2011). Blood viscosity modelling: influence of aggregate network dynamics under transient conditions. Biorheology.

[bib27] Kaliviotis E., Yianneskis M. (2008). Fast response characteristics of red blood cell aggregation. Biorheology.

[bib28] Kim, Y., Kim, K., Park, Y., 2012. Measurement techniques for red blood cell deformability: recent advances, Blood Cell – An Overview of Studies in Hematology

[bib29] Kodali S.K., Williams M.R., Smith C.R., Svensson L.G., Webb J.G., Makkar R.R., Fontana G.P., Dewey T.M., Thourani V.H., Pichard A.D., Fischbein M., Szeto W.Y., Lim S., Greason K.L., Teirstein P.S., Malaisrie S.C., Douglas P.S., Hahn R.T., Whisenant B., Zajarias A., Wang D., Akin J.J., Anderson W.N., Leon M.B., PARTNER Trial Investigators (2012). Two-year outcomes after transcatheter or surgical aortic-valve replacement. N. Engl. J. Med..

[bib30] Leo H., Dasi L., Carberry J., Simon H., Yoganathan A. (2006). Fluid dynamic assessment of three polymeric heart valves using particle image velocimetry. Ann. Biomed. Eng..

[bib31] Lerakis S., Hayek S.S., Douglas P.S. (2013). Paravalvular aortic leak after transcatheter aortic valve replacement: current knowledge. Circulation.

[bib32] Lim A.P.W.L., Chew Y.T., Chew T.C., Low H.T. (1994). Particle image velocimetry in the investigation of flow past artificial heart valves. Ann. Biomed. Eng..

[bib33] Lim W., Chew Y., Chew T., Low H. (1998). Steady flow dynamics of prosthetic aortic heart valves: a comparative evaluation with PIV techniques. J. Biomech..

[bib34] Makkar R.R., Fontana G., Jilaihawi H., Chakravarty T., Kofoed K.F., de Backer O., Asch F.M., Ruiz C.E., Olsen N.T., Trento A., Friedman J., Berman D., Cheng W., Kashif M., Jelnin V., Kliger C.A., Guo H., Pichard A.D., Weissman N.J., Kapadia S., Manasse E., Bhatt D.L., Leon M.B., Søndergaard L. (2015). Possible subclinical leaflet thrombosis in bioprosthetic aortic valves. N. Engl. J. Med..

[bib35] Makkar R.R., Fontana G.P., Jilaihawi H., Kapadia S., Pichard A.D., Douglas P.S., Thourani V.H., Babaliaros V.C., Webb J.G., Herrmann H.C., Bavaria J.E., Kodali S., Brown D.L., Bowers B., Dewey T.M., Svensson L.G., Tuzcu M., Moses J.W., Williams M.R., Siegel R.J., Akin J.J., Anderson W.N., Pocock S., Smith C.R., Leon M.B. (2012). Transcatheter aortic-valve replacement for inoperable severe aortic stenosis. N. Engl. J. Med..

[bib36] Miller D.C., Blackstone E.H., Mack M.J., Svensson L.G., Kodali S.K., Kapadia S., Rajeswaran J., Anderson W.N., Moses J.W., Tuzcu E.M., Webb J.G., Leon M.B., Smith C.R. (2012). Transcatheter (TAVR) versus surgical (AVR) aortic valve replacement: occurrence, hazard, risk factors, and consequences of neurologic events in the PARTNER trial. J. Thorac. Cardiovasc. Surg..

[bib37] Moat N.E., Ludman P., de Belder M.A., Bridgewater B., Cunningham A.D., Young C.P., Thomas M., Kovac J., Spyt T., MacCarthy P.A., Wendler O., Hildick-Smith D., Davies S.W., Trivedi U., Blackman D.J., Levy R.D., Brecker S.J.D., Baumbach A., Daniel T., Gray H., Mullen M.J. (2011). Long-term outcomes after transcatheter aortic valve implantation in high-risk patients with severe aortic stenosis: the U.K. TAVI (United Kingdom Transcatheter Aortic Valve Implantation) Registry. J. Am. Coll. Cardiol..

[bib38] Nombela-Franco L., Webb J.G., de Jaegere P.P., Toggweiler S., Nuis R.-J., Dager A.E., Amat-Santos I.J., Cheung A., Ye J., Binder R.K., van der Boon R.M., Van Mieghem N., Benitez L.M., Perez S., Lopez J., San Roman J.A., Doyle D., DeLarochelliere R., Urena M., Leipsic J., Dumont E., Rodes-Cabau J. (2012). Timing, predictive factors, and prognostic value of cerebrovascular events in a large cohort of patients undergoing transcatheter aortic valve implantation. Circulation.

[bib39] Reul H., Vahlbruch A., Giersiepen M., Schmitz-Rode T., Hirtz V., Effert S. (1990). The geometry of the aortic root in health, at valve disease and after valve replacement. J. Biomech..

[bib40] Reynolds M.R., Magnuson E.A., Wang K., Thourani V.H., Williams M., Zajarias A., Rihal C.S., Brown D.L., Smith C.R., Leon M.B., Cohen D.J. (2012). Health-related quality of life after transcatheter or surgical aortic valve replacement in high-risk patients with severe aortic stenosis: results from the PARTNER (Placement of AoRTic TraNscathetER Valve) Trial (Cohort A). J. Am. Coll. Cardiol..

[bib41] Reynolds W.C., Hussain A.K.M.F. (1971). The mechanism of an organized wave in turbulent shear flow. J. Fluid Mech..

[bib42] Rodés-Cabau J., Dauerman H.L., Cohen M.G., Mehran R., Small E.M., Smyth S.S., Costa M.A., Mega J.L., O’Donoghue M.L., Ohman E.M., Becker R.C. (2013). Antithrombotic Treatment in Transcatheter Aortic Valve Implantation: insights for Cerebrovascular and Bleeding Events. J. Am. Coll. Cardiol..

[bib43] Rodés-Cabau J., Dumont E., Boone R.H., Larose E., Bagur R., Gurvitch R., Bédard F., Doyle D., De Larochellière R., Jayasuria C., Villeneuve J., Marrero A., Côté M., Pibarot P., Webb J.G. (2010). Cerebral embolism following transcatheter aortic valve implantation: comparison of transfemoral and transapical approaches. J. Am. Coll. Cardiol..

[bib44] Samim M., Hendrikse J., van der Worp H.B., Agostoni P., Nijhoff F., Doevendans P. a, Stella P.R. (2015). Silent ischemic brain lesions after transcatheter aortic valve replacement: lesion distribution and predictors. Clin. Res. Cardiol..

[bib45] Smith C.R., Leon M.B., Mack M.J., Miller D.C., Moses J.W., Svensson L.G., Tuzcu E.M., Webb J.G., Fontana G.P., Makkar R.R., Williams M., Dewey T., Kapadia S., Babaliaros V., Thourani V.H., Corso P., Pichard A.D., Bavaria J.E., Herrmann H.C., Akin J.J., Anderson W.N., Wang D., Pocock S.J. (2011). Transcatheter versus surgical aortic-valve replacement in high-risk patients. N. Engl. J. Med..

[bib46] Stühle S., Wendt D., Hou G., Wendt H., Thielmann M., Jakob H., Kowalczyk W. (2011). Fluid dynamic investigation of the ATS 3F enable sutureless heart valve. Innovations (Phila).

[bib47] Swanson W.M., Clark R.E. (1974). Dimensions and geometric relationships of the human aortic value as a function of pressure. Circ. Res..

[bib48] Thubrikar M.J. (1990). The Aortic Valve.

[bib49] Toninato, Salmon, Susin, Ducci, Burriesci, Physiological vortices in the sinuses of Valsalva: an in vitro approach for bio-prosthetic valves, *J. Biomech.* **49**, 2016, 2635–2643 10.1016/j.jbiomech.2016.05.027 PMC5061069 27282961

[bib50] Van Steenhoven A.A., Van Dongen M.E.H. (1979). Model studies of the closing behaviour of the aortic valve. J. Fluid Mech..

[bib51] Vermeer S.E., Longstreth W.T., Koudstaal P.J. (2007). Silent brain infarcts: a systematic review. Lancet Neurol..

[bib52] Webb J., Cribier A. (2010). Percutaneous transarterial aortic valve implantation: what do we know?. Eur. Heart J..

[bib53] Wendt D., Stühle S., Piotrowski J.A., Wendt H., Thielmann M., Jakob H., Kowalczyk W. (2012). Comparison of flow dynamics of Perimount Magna and Magna Ease aortic valve prostheses. Biomed. Tech..

[bib54] Yoganathan A.P., Woo Y.R., Sung H.W. (1986). Turbulent shear stress measurements in the vicinity of aortic heart valve prostheses. J. Biomech..

